# Identification of Transposable Elements Contributing to Tissue-Specific Expression of Long Non-Coding RNAs

**DOI:** 10.3390/genes9010023

**Published:** 2018-01-09

**Authors:** Takafumi Chishima, Junichi Iwakiri, Michiaki Hamada

**Affiliations:** 1 Department of Electrical Engineering and Bioscience, Faculty of Science and Engineering, Waseda University, 55N-06-10, 3-4-1, Okubo Shinjuku-ku, Tokyo 169-8555, Japan; unzncsmtkfm@asagi.waseda.jp; 2 Computational Bio Big-Data Open Innovation Laboratory (CBBD-OIL), National Institute of Advanced Industrial Science and Technology (AIST), 63-520, 3-4-1, Okubo Shinjuku-ku, Tokyo 169-8555, Japan; 3 Graduate School of Frontier Sciences, University of Tokyo, 5-1-5 Kashiwanoha, Kashiwa, 277-8562 Chiba, Japan; iwakiri@cb.k.u-tokyo.ac.jp; 4 Artificial Intelligence Research Center (AIRC), National Institute of Advanced Industrial Science and Technology (AIST), 2-3-26, Aomi, Koto-ku, Tokyo 135-0064, Japan; 5 Institute for Medical-oriented Structural Biology, Waseda University, 2-2, Wakamatsu-cho, Shinjuku-ku, Tokyo 162-8480, Japan; 6 Graduate School of Medicine, Nippon Medical School, 1-1-5, Sendagi, Bunkyo-ku, Tokyo 113-8602, Japan

**Keywords:** long non-coding RNA, transposable element, tissue-specific expression

## Abstract

It has been recently suggested that transposable elements (TEs) are re-used as functional elements of long non-coding RNAs (lncRNAs). This is supported by some examples such as the human endogenous retrovirus subfamily H (HERVH) elements contained within lncRNAs and expressed specifically in human embryonic stem cells (hESCs), as required to maintain hESC identity. There are at least two unanswered questions about all lncRNAs. How many TEs are re-used within lncRNAs? Are there any other TEs that affect tissue specificity of lncRNA expression? To answer these questions, we comprehensively identify TEs that are significantly related to tissue-specific expression levels of lncRNAs. We downloaded lncRNA expression data corresponding to normal human tissue from the Expression Atlas and transformed the data into tissue specificity estimates. Then, Fisher’s exact tests were performed to verify whether the presence or absence of TE-derived sequences influences the tissue specificity of lncRNA expression. Many TE–tissue pairs associated with tissue-specific expression of lncRNAs were detected, indicating that multiple TE families can be re-used as functional domains or regulatory sequences of lncRNAs. In particular, we found that the antisense promoter region of L1PA2, a LINE-1 subfamily, appears to act as a promoter for lncRNAs with placenta-specific expression.

## 1. Introduction

Development of next generation sequencing technology and transcriptome analysis has revealed tens of thousands of long non-coding RNAs (lncRNAs) in the human genome [1,2,3]. lncRNAs are defined as transcripts longer than 200 nt that do not encode proteins [4], many of which are known to be 5’ capped, 3’ polyadenylated [5], and spliced [6] like mRNAs. Only a few of the many lncRNAs have been experimentally characterized, and they are associated with various biological processes, such as chromatin modification and transcriptional regulation, and diseases [4,7,8]. However, only a few percent of lncRNAs are annotated, and the functions of most lncRNAs are still unknown [9].

Thus, the immediate research problem is determining how to elucidate the functions of a large number of unknown lncRNAs efficiently, and understanding the functional elements within lncRNAs will likely help this issue. Like domains or motifs in proteins, lncRNAs should similarly contain RNA elements, such as RNA structures or sequences, fundamental to their various functions [7]. For example, the A-repeat region in Xist, which is an lncRNA responsible for X inactivation in mammals, is essential for chromosomal silencing by Xist [10]. Since little is known about these RNA elements in lncRNAs, common elements among lncRNAs have not yet been detected [7] and prediction of these elements using informatic approaches is required. One of the candidates for lncRNA functional elements consists of transposable elements (TEs), which are abundant within lncRNAs.

TEs are mobile genetic elements with many copies, and they occupy half of the human genome [11]. Among TEs within the human genome, retrotransposed TE copies comprise the majority, and they can be classified into three major types: long interspersed elements (LINEs), short interspersed elements (SINEs), and LTR retrotransposons [11]. LINEs are about 6 kb long and are transposed by the proteins encoded within their own sequences [11]. In contrast, SINEs are short (about 100–400 bp) and encode no proteins [11]. Most of them share their 3’ ends with LINEs and are thought to be transposed by the LINE machinery [11]. LTR retrotransposons are characterized by two long terminal repeats (LTRs) flanking their coding sequences. They are regulated by several regulatory elements within their LTRs and are transposed by the proteins encoded in their coding sequences [11]. Endogenous retroviruses (ERV) are included within this type of TEs. While these TEs are usually regarded as selfish genes, sequences derived from TEs have sometimes been found to be re-used by host organisms and this phenomenon is called exaptation. In one notable example, the env proteins encoded by an endogenous retrovirus like those of human endogenous retrovirus subfamily W (HERVW) plays a role in mammalian placenta formation [12]. It is also known that the regulatory sequence of some TEs are still active and can be used as alternative promoters of host coding genes [13,14].

In recent years, TEs have been discovered to be remarkably enriched within lncRNA exons relative to protein-coding gene exons [15,16], and the hypothesis that TEs serve as one of the functional elements in lncRNAs has thus attracted the attention of researchers [17]. One example already found involves 1/2-sbsRNA, in which an Alu element recognizes another complementary Alu element in a mRNA and induces its degradation [18]. In another example, lncRNAs containing HERVH elements are expressed specifically in human embryonic stem cells (hESCs) [15] and are required to maintain hESC identity [19]. The LTR region of HERVH elements acts as an enhancer and induces stem cell-specific expression of surrounding genes [19]. Moreover, the transcribed HERVH-lncRNAs interact with pluripotency-related factors such as OCT4, suggesting that they act as a scaffold, recruiting those factors to HERVH LTR regions [19]. While multiple examples have been reported, it is still unknown how widely TEs are re-used among all lncRNAs.

In order to elucidate this issue, we focused on the tissue specificity of lncRNAs and comprehensively identified the TEs that affect the tissue specificity of lncRNAs in normal human tissues. Many TE–tissue pairs were detected, indicating that multiple TE families are re-used as functional domains or regulatory sequences of lncRNAs. In particular, for L1PA2, a subfamily of LINE-1, our results suggested that its antisense promoter region acts as promoter producing the lncRNAs that are expressed specifically in placenta tissue.

## 2. Materials and Methods

### 2.1. Input Data

Annotations of lncRNAs and protein-coding genes were obtained from GENCODE v24 (https://www.gencodegenes.org/), and only the longest transcript was selected for each gene with several transcripts. As the TE annotations, we used the mapping result of Repeat Library 20140131 to hg38, which is published by RepeatMasker [20], after excluding simple repeats, low-complexity, non-coding RNA, and satellites. Overlaps between lncRNAs and TEs were detected using our own Perl script. Only exonic regions of lncRNAs were considered. Three out of the four previously used gene expression datasets [21] were utilized again (Table 1). These are mainly based on normal human tissue RNA-seq data, which were obtained from Expression Atlas (https://www.ebi.ac.uk/gxa/home) [22]. As the remaining dataset (produced by the Epigenome Roadmap Project, http://www.roadmapepigenomics.org/) was derived from fetal tissues, it was excluded from this analysis. Since tissues in the Genotype-Tissue Expression (GTEx) data were classified more finely than those in other datasets and cultured cells were included, only 30 normal tissues from this dataset were analyzed as in the previous study [21].

### 2.2. Identification of Transposable Elements that Contribute to Tissue-Specific Expression of Long Non-Coding RNAs

**Figure 1:**
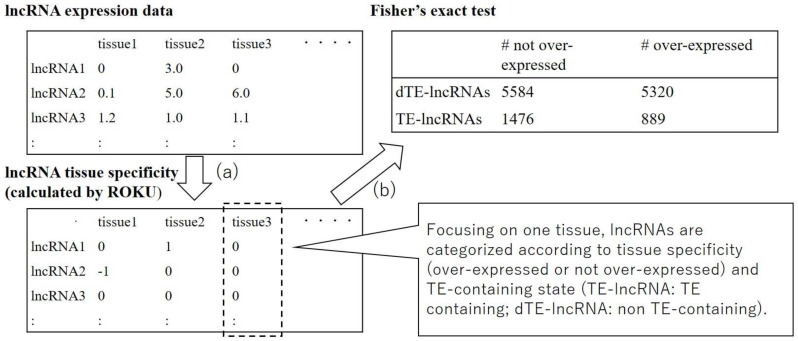
The flow of the analysis. (**a**) First, expression levels are converted into tissue specificity by ROKU. (**b**) Then, the results for each tissue were aggregated separately for long non-coding RNAs (lncRNA) containing a specific transposable element (TE; shown as TE-lncRNA) and for lncRNA not containing that specific TE (shown as dTE-lncRNA), and the significance of the difference between lncRNAs in these categories was determined using Fisher’s exact tests.

Based on the obtained data (in Section 2.1), we identified TEs that contribute to tissue-specific expression of lncRNAs. The process was mainly divided into the calculation of the tissue specificity of lncRNAs and the statistical test determining whether the presence or absence of each TE-derived sequence influences the tissue specificity of lncRNA expression (Figure 1). First, the tissue specificity of lncRNAs was calculated from gene expression data using ROKU [26] as in the previous research [21]. ROKU is a program that detects outliers from the multiple tissue expression level estimates for each gene. It returns 1, −1, or 0 when the expression level is specifically increased in the tissue, specifically decreased in the tissue, or not tissue specific, respectively. In order to limit the influence of transcriptional noise, filtering was performed in which the tissue specificity was converted to 0 (not specific) if the corresponding expression level was lower than 1 fragment per kilobase of transcript per million mapped reads (FPKM). After calculating tissue specificity, statistical tests were performed exhaustively in order to investigate whether the presence of a specific TE in lncRNAs affects the tissue specificity of lncRNAs. In detail, we examined whether the proportion of the genes whose expression was specifically increased in a specific tissue differed among the lncRNAs containing the TE of interest and the lncRNAs that do not contain the TE by using Fisher’s exact tests. This test was conducted for all TE–tissue pairs and the results were obtained in the list of pairs of the TE and the related tissue. We conducted the above analysis under 2 × 2 conditions concerning TE orientation with respect to lncRNAs (considering only sense-oriented TEs/only antisense-oriented TEs) and TE classification level (TE family level/TE subfamily level). The *p*-values of these statistical tests were corrected using the Benjamini–Hochberg method [27] for each condition, and only TE–tissue pairs with corrected *p*-value < 0.05 were considered significant.

## 3. Results

### 3.1. Multiple Transposable Element Families Are Significantly Related to Tissue-Specific Expression of Long Non-Coding RNAs

We conducted separate analyses for each TE family and for each TE subfamily. In the following results, lncRNAs with a specific TE (e.g., Alu) are referred to as TE-lncRNAs (e.g., Alu-lncRNAs), and lncRNAs without the specific TE are referred to as dTE-lncRNAs (e.g., dAlu-lncRNAs).

#### 3.1.1. Transposable Element Family-Level Analysis

**Figure 2:**
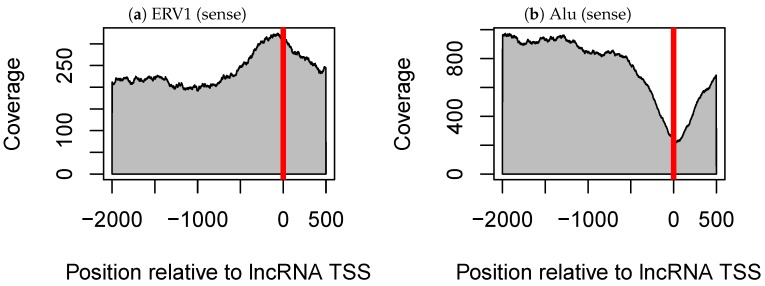
Coverage of (**a**) ERV1 elements and (**b**) Alu elements around transcription start site (TSS) in long non-coding RNAs (lncRNAs), where ERV1 and Alu elements with the same orientation as their corresponding lncRNAs are considered. In each figure panel, the horizontal axis shows the relative position with respect to lncRNA TSSs (where 0 indicates the TSSs), and the vertical axis shows the coverage of the transposable element.

Many TE–tissue pairs were detected, including two pairs that were common to analyses of the three datasets (Table 2). The first one was the pair of ERV1 elements and testis tissue (5–7 in Table 2). ERV1-lncRNAs were likely to be expressed specifically in testis tissue, compared with dERV1-lncRNAs (lncRNA without ERV1). It is known that the LTR region of ERV elements regulates the expression of protein coding genes [13]. Kelley and Rinn suggested that ERV LTRs also regulated the expression of lncRNAs, as ERV sequences were enriched at the transcription start sites (TSSs) of lncRNAs and tended to be in the same orientation as those lncRNAs [15]. We also observed the enrichment of ERV1 within lncRNA TSSs using our dataset (Figure 2a). ERV1 enrichment in lncRNA TSSs and the pairing of ERV1 and testis were detected only when ERV1 sequences were oriented in the same direction as their corresponding lncRNAs (Figure 2a and Supplementary Figure S1a). These results further support the hypothesis that ERV1 LTR regulates the expression of lncRNAs.

The other pair observed among all three datasets consisted of Alu and testis (15–17 in Table 2). However, Alu-lncRNAs were less likely to be expressed specifically in testis tissue compared with dAlu-lncRNAs, which is consistent with the results of a previous study in which Alu-lncRNAs were observed to be highly expressed in all tissues except testis tissue [15]. We confirmed this increased expression and found that expression of Alu-lncRNA in all tissues other than testis tissue increased at a constant rate with respect to dAlu-lncRNA (Supplementary Figure S2). Unlike ERV1, Alu is distributed away from lncRNA TSSs (Figure 2b and Supplementary Figure S1b). Interestingly, the pair of Alu and testis tissue was detected regardless of the orientation of Alu elements relative to the lncRNAs. We considered that only Alu elements oriented in one direction had a true effect and that a significant difference was detected for Alu elements in both directions owing to the influence of lncRNAs having a pair of Alu elements in both directions (i.e., inverted Alu elements). However, this hypothesis was not supported, and both Alu elements in the sense and antisense orientations relative to lncRNAs influence the testis specificity of lncRNAs. We divided Alu-lncRNAs into three classes (containing only sense Alu, containing only antisense Alu and containing one or more pairs of inverted Alu elements), examined the difference in tissue specificity with dAlu-lncRNAs again and confirmed that all classes were significantly different from dAlu-lncRNAs (Supplementary Table S1).

#### 3.1.2. Transposable Element Subfamily-Level Analysis

In the subfamily level analyses, no TE–tissue pairs were shared among each of the three datasets (Table 3). The pair of Alu subfamilies and adrenal tissue was detected in the Illumina body map data (1–6, 8, 10, 11 in Table 3). Alu-lncRNAs were likely to be expressed specifically in adrenal tissue relative to dAlu-lncRNAs. However, this pair was not found in the other datasets, though all the datasets contained adrenal tissue, suggesting that the Illumina body map samples may have been collected under abnormal conditions (such as a disease). The pair of L1PA2 and placenta tissue was detected in the Human Protein Atlas data (12 in Table 3). L1PA2-lncRNAs were likely to be expressed specifically in placenta tissue relative to dL1PA2-lncRNAs. Since only the Human Protein Atlas data contained placenta samples, the reproducibility of this result could not be verified. Even so, it is notable that the result was detected only when L1PA2 was oriented in the opposite direction relative to lncRNAs, and we thus performed further analyses to assess this result (Section 3.3).

### 3.2. The Alu Family Is Significantly Related not only to Tissue-Specific Expression of Long Non-Coding RNAs but also to that of mRNAs

To examine whether the relation between TEs and gene regulation changes between lncRNAs and mRNAs, we performed a homologous analysis for mRNAs. The procedure is the same as that described above except that mRNAs were used as input data and UTRs (untranslated regions) were screened to judge overlap with TEs. As we have analyzed lncRNAs, many TE–tissue pairs were detected (Table 4 and Table 5). Although no pairs were discovered to be common to all three datasets, lncRNAs with DNA transposon hAT-Charlie or Alu or MIR were less likley to be expressed specifically in testis tissue, which was common to Illumina Body Map and Human Protein Atlas datasets (5, 6, 25, 26, 32, 33 in Table 4). In the detected pairs, only the Alu–testis pair was common with lncRNAs (25, 26 in Table 4). Interestingly, though the pair of ERV1 (+ strand) and testis was detected (14 in Table 4), the effect was opposite (i.e., expression was less tissue specific in the case of mRNAs).

### 3.3. The Antisense Promoter of L1PA2 May Contribute to Placenta-Specific Transcription of Long Non-Coding RNAs

In order to investigate the mechanism by which lncRNAs containing specific TEs were likely to be expressed in specific tissues, we focused on L1PA2 identified from the results of the above analysis. L1PA2-lncRNAs were likely to be expressed specifically in placenta tissue (12 in Table 3). L1PA2 is a evolutionary recent TE subfamily belonging to LINE 1, with about 5000 copies in the human genome. There are 50 copies of L1PA2 in lncRNAs and, notably, 33 of the copies exist at TSSs and in an antisense orientation (Supplementary Table S2). Furthermore, many studies have reported that L1 has an antisense promoter (ASP) region near its TSS, which drives transcription in a direction opposite to that of L1PA2 and produces transcripts from upstream proximal regions [28,29,30,31]. Therefore, we hypothesized that the ASP region of L1PA2 functions as a promoter of lncRNAs and drives placenta-specific expression of L1PA2-lncRNAs. In order to verify this hypothesis, an additional analysis was performed.

#### 3.3.1. Antisense L1PA2 Was Enriched in Long Non-Coding RNA TSSs and Overlapped with lncRNAs by Approximately 500 nt

**Figure 3:**
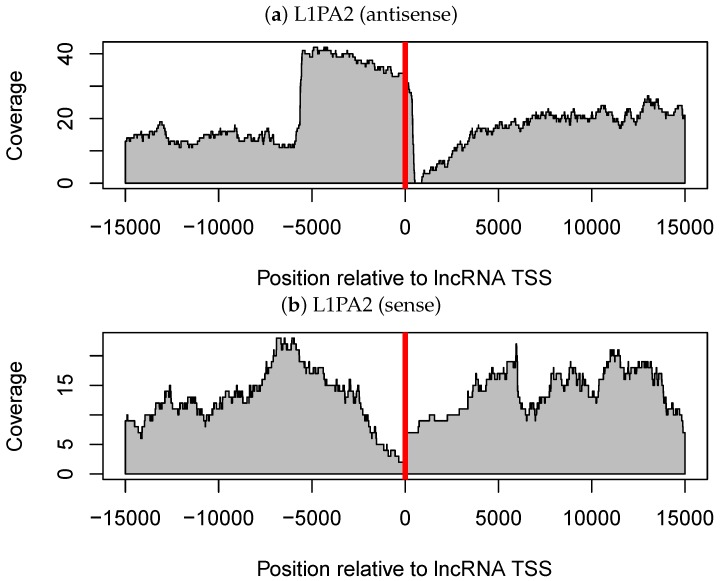

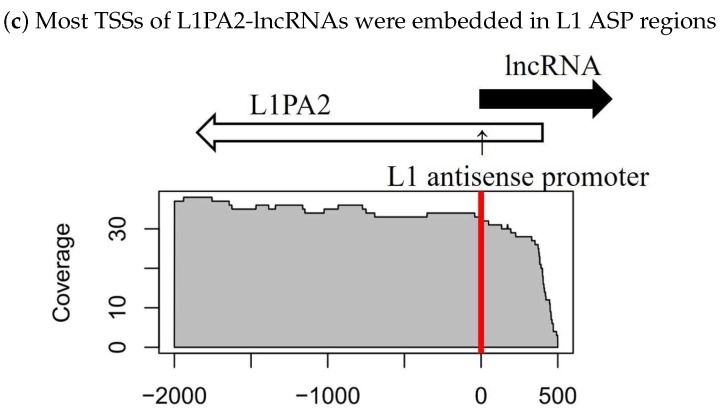
Coverage of L1PA2 elements around the transcription start site (TSSs) of long non-coding RNAs (lncRNAs). (**a**) L1PA2 elements in the same orientation as the lncRNAs are considered. (**b**) L1PA2 elements in the opposite orientation relative to the lncRNAs are considered. (**c**) The region around the TSSs in (**a**) is enlarged (showing greater detail between positions −2000 and 500 in (**a**)). In each panel, the horizontal axis shows the relative position with respect to lncRNA TSSs (where 0 indicates TSSs), and the vertical axis shows the coverage of the transposable element.

We investigated a general trend in the location of L1PA2 elements relative to lncRNA TSSs. Notably, most of the antisense L1PA2 elements were located at nearly the same site relative to lncRNA TSSs. These L1PA2 elements overlapped with lncRNAs by about 500 nt (Figure 3a,c). In other words, most TSSs of L1PA2-lncRNAs were embedded in L1 ASP regions, which are located in the L1 5’-UTR (positions 400–600) [29]. Furthermore, as we expected, this trend was not observed in L1PA2 elements in a sense orientation relative to lncRNAs (Figure 3b). Thus, these findings support the hypothesis that the ASP region of L1PA2 functions as a promoter of lncRNAs. Indeed, transcripts derived from L1 ASP were comprehensively identified by Criscione et al. 2016 [31]. However, among the 33 lncRNAs detected in this study (Supplementary Table S2), 23 out of the 33 lncRNAs were newly identified by the present analyses.

#### 3.3.2. The Level of H3K4me3 in L1PA2- Long Non-Coding RNAs Regions Increased Specifically in Placenta Tissue

**Figure 4:**
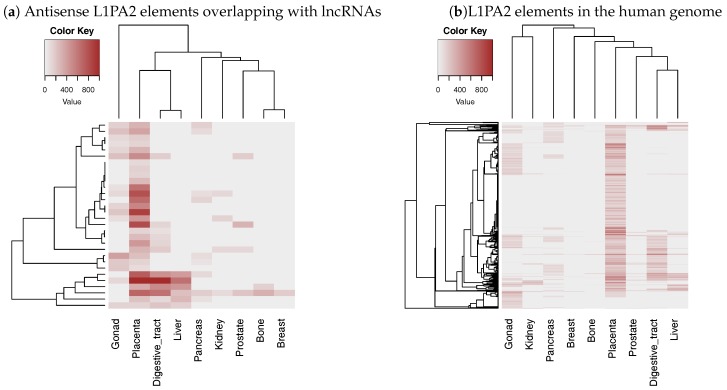
The H3K4me3 histone modification level in the 5’ regions of L1PA2 elements (positions 0–1000). Each rows represents a L1PA2 element, and each columns represent a sample. Only L1PA2 elements overlapping peaks in one or more tissues are shown. Samples in which all L1PA2 elements did not overlap with peaks were excluded from the figure. The intensity of the color of each cell indicates the maximum value of the peak score (−10log(*Q*-value)) within the 5’ region for a L1PA2 element. (If there are no peaks in the region, the score is 0.) (**a**) Only L1PA2 elements overlapping with long non-coding RNA (lncRNA) TSSs in the opposite orientation were considered. (**b**) L1PA2 elements in the human genome including those not overlapping with any lncRNAs were considered.

In order to investigate whether the L1PA2 ASP region contributes to the placenta specific-expression of lncRNAs, we investigated histone modification levels of L1PA2-lncRNAs. We obtained an H3K4me3 histone modification data file (His.ALL.05.H3K4me3.AllCell.bed^1^) containing peak coordinates and scores (−10log(*Q*-value)) from ChIP-Atlas (http://chip-atlas.org/). Since the obtained data is based on hg19, we used GENCODE v24 (mapped to GRCh37) and the mapping result of Repeat Library 20140131 to hg19, which is published by RepeatMasker, as annotations of lncRNAs and TEs, respectively, in this analysis. We selected 15 samples derived from normal tissues or cell lines (Supplementary Table S3) and calculated the maximum peak score in the 5’ regions of L1PA2 elements (positions 0–1000) for each sample. This analysis revealed that H3K4me3 histone modification levels, which are an indicator of transcriptional activity, increased specifically in placenta sample for L1PA2 elements in the antisense orientation relative to their corresponding lncRNAs and overlapping with the TSSs of lncRNAs (Figure 4a). Thus, placenta-specific activation of L1PA2-lncRNA was confirmed by both expression level and histone modification. Interestingly, placenta-specific modifications were also observed for L1PA2 elements in the human genome, including those not overlapping with any lncRNAs (Figure 4b). This suggests that L1PA2 itself may have some undiscovered features that cause placenta-specific activation such as transcription factor binding motifs.

## 4. Discussion

Transposable elements are attracting broad research attention as one of the functional domains of lncRNAs [17]. In this study, we identified TEs related to tissue-specific expression of lncRNAs, and it was confirmed that multiple TEs were associated with tissue specificity of lncRNAs. Two TE–tissue pairs related to tissue-specific expression of lncRNAs were well confirmed.

The first pair consisted of ERV1 elements and testis tissue. ERV1-lncRNAs (i.e., lncRNAs including ERV1) were likely to be expressed specifically in testis. This was observed only when ERV1 was oriented in the same direction as lncRNAs. Further, ERV1 elements were enriched at the TSSs of lncRNAs, suggesting that the LTRs of ERV1 elements act as regulatory sequences for lncRNAs and contribute to testis-specific expression of lncRNAs. This is supported by several previous research. First, many studies have reported that TEs, including ERV, provide regulatory sequences and contribute to the transcriptional regulation of host genes [13,14,32]. Kelley and Rinn previously proposed that ERV elements contribute to the transcriptional regulation of lncRNAs, based on ERV enrichment at lncRNA TSSs and the tendency of ERV elements to be sense oriented relative to lncRNA TSSs [15]. Moreover, a study conducted as part of the FANTOM4 project showed that transcripts derived from retrotransposons have tissue-specific expression, and ERV1-derived transcripts are expressed in a testis-specific manner [14]. On the other hand, it was slightly surprising that TEs acted as regulatory sequences because we expected that TEs mainly acted as a functional domain on transcribed RNA and contributed to post-transcriptional regulation.

The other TE–tissue pair consisted of Alu elements and testis tissue. Alu-lncRNAs were less likely to be expressed specifically in testis tissue. This observation was also detected in mRNAs, suggesting that there may exist some common mechanisms between mRNAs and lncRNAs. As Alu elements were distributed to avoid lncRNA TSSs (cf. Figure 2), Alu elements may be act as sequence and/or structure motif in the transcribed RNAs rather than as a regulatory sequence in DNA. There are two candidates for specific mechanisms that reduce testis-specific expression of Alu-lncRNAs, including ADAR-mediated RNA editing and PIWI-interacting RNA (piRNA)-mediated gene silencing. ADAR is an enzyme that causes adenosine to inosine editing, and it mainly targets double-stranded RNA (dsRNA) formed by inverted pairs of Alu elements [33]. Alu elements contained within lncRNAs can form intramolecular stem-loop structures or long intermolecular dsRNAs [34] and be targeted by ADAR. Although the function of the edited lncRNA is not well understood, it has been proposed that the edited lncRNA is degraded by Tudor-SN [34]. Accordingly, ADAR may contribute to the regulation of Alu-lncRNA expression. This hypothesis is supported by the testis specificity of lncRNAs being more affected in lncRNAs, including inverted Alu pairs, compared to lncRNAs containing Alu elements in only one orientation (Supplementary Table S1). However, ADAR1 and ADAR2 are expressed in various tissues [33], and these alone cannot explain the testis specificity. The testis specificity may be explained by piRNA-mediated gene silencing. PIWI-interacting RNAs are a germ cell-specific small RNAs that forms a complex with PIWI proteins and induces gene silencing [35,36]. piRNAs recognize the target genes based on complementarity between the target transcripts and themselves [36]. Since many piRNAs are derived from antisense transposons, they can silence sense transposon-derived transcripts [36]. One recent study shows that lncRNAs expressed in the testis are degraded by the piRNA pathway [37]. Thus, piRNA-mediated silencing can explain the decreased testis specificity of Alu-lncRNAs. In addition, the pair of Alu and testis tissue is observed regardless of the orientation of TEs with respect to lncRNAs. In general, the piRNA pathway is thought to mainly silence transcripts containing TEs in the sense direction, but our result may saggest that also lncRNAs containing antisense TEs are downregulated by piRNAs at the same time. However, further experiments and analyzes on piRNAs are required to verify this.

Finally, at the TE subfamily level, we found that L1PA2-lncRNAs were more likely to be expressed in placenta tissue. Our detailed follow-up analysis on this suggested that L1PA2 antisense promoter (ASP) regions probably act as lncRNA promoters and contribute to tissue-specific transcription of lncRNAs, which was confirmed by the location of L1PA2 elements relative to lncRNA TSSs and histone modification of L1PA2-lncRNAs. Furthermore, since L1PA2 is activated in placenta tissue regardless of its overlap with lncRNAs, some sequence elements regulating placenta specificity should exist in L1PA2 elements themselves. We are still searching for these elements using sequence homology search and transcription factor motif search. Placenta-specific activation of the L1 elements was not observed in the older L1PA4 and L1PA5 subfamilies, but it is observed in more recent subfamilies than L1PA4 (Supplementary Figure S3). Thus, these elements contribute to placenta specific expression of lncRNAs should consist of new sequences obtained after the origin of L1PA3 elements.

In this study, we clarified that multiple TEs affect tissue specificity of lncRNAs, but the functions of those lncRNAs are still unknown. One possibility is their functions are related to piRNAs. Recent studies have revealed that a certain number of lncRNAs act as piRNA precursors [38,39]. In our study, ERV1-lncRNAs tend to show testis-specific expression and may act as piRNA precursors. Moreover, it has been reported that ERV elements are concentrated in lncRNAs that act as piRNA precursors relative to the genomic background [39]. On the other hand, as mentioned above, some TE-lncRNAs such as Alu-lncRNAs may be the targets of piRNAs. In any case, in order to elucidate the functions of these TE-lncRNAs, additional studies employing experimental approaches including knockdown or comprehensive analysis of interactions between the lncRNAs and other elements are required.

In this study, we examined the distribution of TEs near the TSSs of lncRNAs, but it is also important to examine the distribution of TEs in other regions of lncRNAs such as the 3’ ends or the inside regions of the transcripts. In the past, the FANTOM project reported that human and mouse RefSeq transcripts containing TEs in their 3’ UTRs had lower expression levels than transcripts without TEs in the 3’ UTRs [14]. On the other hand, Kelley and Rinn reported that Alu elements had a peak approximately 250 bases downstream of the 3’ ends of lncRNAs and presumably functioned as polyadenylation signals for lncRNAs [15]. It is interesting whether such TEs also affect the tissue specificity of lncRNAs. Moreover, although we focused only on the tissue specificity of lncRNAs in this study, it is worth investigating the relation between other properties of lncRNAs and TEs. Localization of lncRNAs and their interactions with other molecules are considered to be very important clues to their function [8]. Several studies have suggested that TEs also have important roles in localization and intermolecular interactions with lncRNAs, by acting as the recognition domain within lncRNAs [40,41,42].

## 5. Conclusions

In this study, we comprehensively identified the TEs that are associated with the tissue specificity of lncRNAs expression. Many TE–tissue pairs were detected, suggesting that many TEs may be re-used for regulation of lncRNAs. In particular, the L1PA2 antisense promoter region probably acts as a promoter of lncRNAs and drives tissue-specific expression of lncRNAs. These results strengthen the hypothesis that TEs serve as one of the functional elements in lncRNAs.

## Figures and Tables

**Table 1:** Gene expression datasets obtained from Expression Atlas.

ID	Expression Atlas ID	Data Provider	# Tissues	# Samples	Reference
1	E-MTAB-513	Illumina Body Map	16	19	[23]
2	E-MTAB-2836	Human Protein Atlas	32	122	[24]
3	E-MTAB-2919	Genotype-Tissue Expression (GTEx)	53	3282	[25]

ID is used to refer to each of the three datasets in Table 2, Table 3, Table 4 and Table 5. Respectively, # tissues and # samples indicate the number of tissues and samples in each dataset.

**Table 2:** TE families significantly related to tissue-specific expression of long non-coding RNAs (lncRNAs).

No.	TE Family	Tissue	Strand	Effect	Data ID
1	LINE.L1	Brain	−	more specific	1
2	LINE.L1	Cerebral_cortex	−	more specific	2
3	LTR.ERV1	Leukocyte	+	more specific	1
4	LTR.ERV1	Placenta	+	more specific	2
5	LTR.ERV1	Testis	+	more specific	1
6	LTR.ERV1	Testis	+	more specific	2
7	LTR.ERV1	Testis	+	more specific	3
8	LTR.ERVL	Bone_marrow	+	less specific	2
9	LTR.ERVL.MaLR	Bone_marrow	+/−	less specific	2
10	SINE.Alu	Adrenal	+/−	more specific	1
11	SINE.Alu	Bone_marrow	+	more specific	2
12	SINE.Alu	Brain	+	more specific	3
13	SINE.Alu	Lymph_node	−	more specific	1
14	SINE.Alu	Skin	+/−	more specific	2
15	SINE.Alu	Testis	+/−	less specific	1
16	SINE.Alu	Testis	+/−	less specific	2
17	SINE.Alu	Testis	+/−	less specific	3

A list of transposable element (TE) families related to tissue specificity of lncRNAs is shown. Strand indicates the orientation of the TE relative to the lncRNAs: +, relations were detected only when TEs were sense relative to lncRNAs; −, relations were detected only when TEs were antisense relative to lncRNAs; +/−, relations were detected when TEs are in both sense and antisense orientations relative to lncRNAs. Effect indicates whether lncRNAs including TEs (i.e., TE-lncRNAs) tended to be expressed specifically in that tissue: more specific, TE-lncRNAs were likely to be expressed specifically in that tissue; less specific, TE-lncRNAs were less likely to be expressed specifically in that tissue. Data id refers to dataset IDs provided in Table 1.

**Table 3:** TE subfamilies significantly related to tissue-specific expression of long non-coding RNAs (lncRNAs).

No.	TE Subfamily	Tissue	Strand	Effect	Data ID
1	AluJb	Adrenal	−	more specific	1
2	AluSc	Adrenal	+/−	more specific	1
3	AluSg	Adrenal	−	more specific	1
4	AluSp	Adrenal	+/−	more specific	1
5	AluSq2	Adrenal	−	more specific	1
6	AluSx	Adrenal	+/−	more specific	1
7	AluSx	Testis	+/−	less specific	1
8	AluSx1	Adrenal	+/−	more specific	1
9	AluSx1	Testis	+	less specific	1
10	AluSz	Adrenal	+/−	more specific	1
11	AluY	Adrenal	+	more specific	1
12	L1PA2	Placenta	−	more specific	2

A list of transposable element (TE) subfamilies related to tissue specificity of lncRNAs is shown. For a detailed explanation of each column, see the caption for Table 2. Placenta samples were included only in the Human Protein Atlas (data ID: 2).

**Table 4:** TE families significantly related to tissue-specific expression of mRNAs.

No.	TE Family	Tissue	Strand	Effect	Data ID
1	DNA	Brain	−	more specific	1
2	DNA.TcMar.Tigger	Testis	−	less specific	3
3	DNA.hAT.Blackjack	Lung	−	more specific	3
4	DNA.hAT.Charlie	Brain	−	more specific	1
5	DNA.hAT.Charlie	Testis	+	less specific	1
6	DNA.hAT.Charlie	Testis	+	less specific	2
7	DNA.hAT.Charlie	Thyroid	+	more specific	1
8	LINE.CR1	Brain	+/−	more specific	1
9	LINE.CR1	Cerebral_cortex	+/−	more specific	2
10	LINE.CR1	Kidney	+	more specific	1
11	LINE.L2	Brain	−	more specific	1
12	LINE.L2	Gall_bladder	+	more specific	2
13	LINE.L2	Ovary	+	more specific	1
14	LTR.ERV1	Testis	+	less specific	1
15	LTR.ERVK	Liver	−	more specific	1
16	LTR.ERVL	Skeletal_muscle	+	less specific	1
17	LTR.Gypsy	Brain	+	more specific	1
18	RC..Helitron.	Heart	+	more specific	1
19	SINE.Alu	Esophagus	−	less specific	2
20	SINE.Alu	Lung	+/−	less specific	1
21	SINE.Alu	Lymph_node	−	less specific	1
22	SINE.Alu	Minor_salivary_gland	+	less specific	3
23	SINE.Alu	Salivary_gland	+	less specific	2
24	SINE.Alu	Stomach	+	less specific	2
25	SINE.Alu	Testis	+/−	less specific	1
26	SINE.Alu	Testis	+/−	less specific	2
27	SINE.MIR	Brain	+/−	more specific	1
28	SINE.MIR	Brain	+/−	more specific	3
29	SINE.MIR	Cerebral_cortex	+/−	more specific	2
30	SINE.MIR	Ovary	+/−	more specific	1
31	SINE.MIR	Prostate	+	more specific	1
32	SINE.MIR	Testis	+/−	less specific	1
33	SINE.MIR	Testis	−	less specific	2

A list of TE families related to tissue specificity of mRNA expression is shown. For a detailed explanation of each column, see the caption in Table 2.

**Table 5:** TE subfamilies significantly related to tissue-specific expression of mRNAs.

No.	TE Subfamily	Tissue	Strand	Effect	Data ID
1	MIR3	Brain	+/−	more specific	1
2	MIR3	Testis	−	less specific	2
3	MIRc	Brain	+/−	more specific	1
4	MIRc	Cerebral_cortex	−	more specific	2
5	MIRc	Ovary	−	more specific	1
6	MamGyp.int	Brain	+	more specific	1

A list of TE subfamilies related to tissue specificity of mRNAs is shown. For a detailed explanation of each column, see the caption in Table 2.

## Footnotes

1. This bed file was obtained from the following url: http://dbarchive.biosciencedbc.jp/kyushu-u/hg19/assembled/His.ALL.05.H3K4me3.AllCell.bed (ChIP-Atlas v7, accessed on Jul 31st, 2017).

## Supplementary Materials

The following are available online at www.mdpi.com/2073-4425/9/1/23/s1. Figure S1: Coverage of ERV1 and Alu elements around transcription start sites (TSSs) of long non-coding RNAs (lncRNAs), where ERV1 and Alu elements with antisense orientations relative to lncRNAs are considered, Figure S2: A comparison between expression levels of long non-coding RNAs (lncRNAs) including Alu elements (Alu-lncRNAs) and those of lncRNAs not containing Alu elements (dAlu-lncRNAs), Figure S3: The H3K4me3 histone modification level of the each L1 subfamily transposable elements (TEs) closely related to L1PA2, Table S1: Alu elements in both the sense and antisense directions relative to long non-coding RNAs (lncRNAs) reduced testis specificity of lncRNA expression, Table S2: A list of transcribed lncRNAs including antisense L1PA2 elements at the 5’ ends, Table S3: Samples used in the ChIP-seq analysis.
