# Supplementary material for: Identification of Transposable Elements Contributing to Tissue-Specific Expression of Long Non-Coding RNAs
Source: Genes (Basel). 2018 Jan 9;9(1):23. doi: 10.3390/genes9010023 (PMC5793176; doi:10.3390/genes9010023)
Supplement: Supplementary file 1 [file genes-09-00023-s001.pdf]

Supplementary Materials for “Identification of transposable elements contributing to tissue-specific expression of long non-coding RNAs”

Takafumi Chishima, Junichi Iwakiri and Michiaki Hamada*

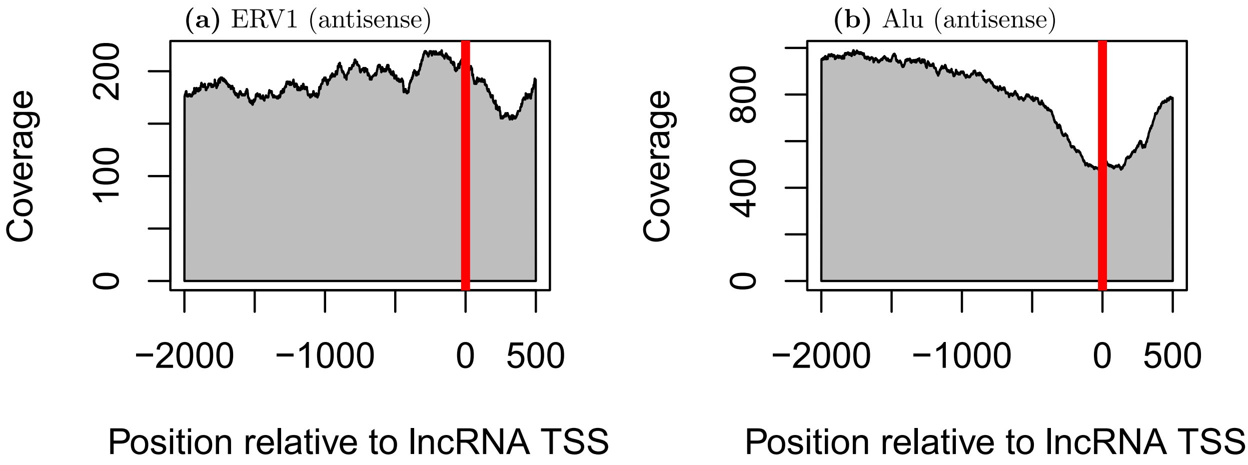

Figure S1: Coverage of (**a**) ERV1 and (**b**) Alu elements around transcription start sites (TSSs) of long non-coding RNAs (lncRNAs), where ERV1 and Alu elements with antisense orientations relative to lncRNAs are considered. In each figure, the horizontal axis shows the relative position with respect to lncRNA TSSs (where 0 indicates TSSs), and the vertical axis shows the coverage of the transposable element.

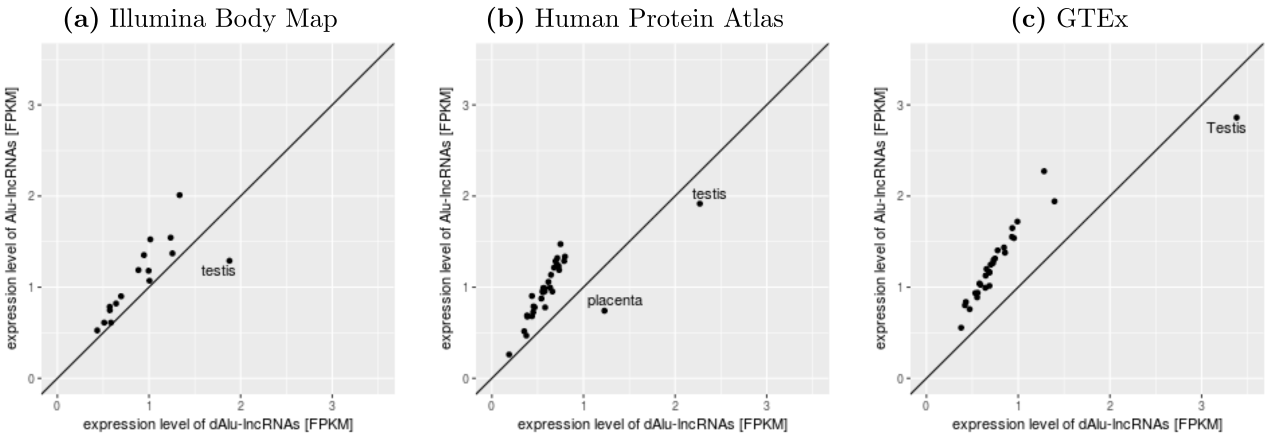

Figure S2: A comparison between expression levels of long non-coding RNAs (lncRNAs) including Alu elements (Alu-lncRNAs) and those of lncRNAs not containing Alu elements (dAlu-lncRNAs). Results are shown for each of the three expression datasets (see in Table 1). Each plot in the figure represents the average expression level of lncRNAs in a specific tissue. When calculating the average of the expression levels, lncRNAs with low expression levels (lower than 1 FPKM) in all the tissues were excluded.

*To whom correspondence should be addressed. Tel: +81 3 5286 3130; Fax: +81 3 5286 3130; Email: mhamada@waseda.jp

Table S1: Alu elements in both the sense and antisense directions relative to long non-coding RNAs (lncRNAs) reduced testis specificity of lncRNA expression

(a) Illumina Body Map

Alu insertion in lncRNA	# not over-expressed	# over-expressed	Ratio	p-value
none(control)	10010	1383	-	-
sense	1405	101	0.55	5.3e-11
antisense	1936	159	0.63	3.9e-10
inverted	776	29	0.30	1.3e-16

(b) Human Protein Atlas

Alu insertion in lncRNA	# not over-expressed	# over-expressed	Ratio	p-value
none(control)	9133	2260	-	-
sense	1281	225	0.75	3.7e-06
antisense	1779	316	0.76	2.2e-07
inverted	732	73	0.46	5.8e-16

(c) GTEx

Alu insertion in lncRNA	# not over-expressed	# over-expressed	Ratio	p-value
none(control)	7757	2809	-	-
sense	1023	279	0.81	5.0e-05
antisense	1491	415	0.82	8.0e-06
inverted	602	91	0.49	8.2e-17

These tables show the relation between the insertion pattern of Alu elements in lncRNAs and the specificity of lncRNA expression in testis tissue. The results are shown for each of the three expression datasets (see in Table 1). # over-expressed indicates the number of lncRNAs whose tissue specificity as calculated by ROKU is 1. # not over-expressed indicates the number of lncRNAs whose tissue specificity as calculated by ROKU is -1 or 0. Ratio is [# over-expressed / # not over-expressed] over [# over-expressed / # not over-expressed] in the control. These p-values werecalculated using Fisher's exact tests.

Table S2: A list of transcribed lncRNAs including antisense L1PA2 elements at the 5' ends

**Chr**	**Gene Id**	**Transcript Id**	**LncRNA**			**L1PA2**		
			**Start**	**End**	**Strand**	**Start**	**End**	**Strand**
chr1	ENSG00000225087.1	ENST00000445976.1	72748921	72899140	−	72898769	72900480	+
chr2	ENSG00000222017.1	ENST00000409845.1	197693106	197774823	+	197687547	197693561	−
chr2	ENSG00000197585.9	ENST00000437883.1	214311602	214684246	−	214683797	214689805	+
chr2	ENSG00000235726.5	ENST00000413842.1	234882279	234888802	−	234888390	234894419	+
chr3	ENSG00000189229.10	ENST00000433639.1	6490479	6736129	+	6484912	6490929	−
chr3	ENSG00000237978.5	ENST00000437488.5	178526505	178860352	−	178859949	178865979	+
chr4	ENSG00000249413.2	ENST00000508572.1	65998846	66150012	+	65993300	65999302	−
chr5	ENSG00000249894.1	ENST00000514791.1	67800740	67890096	−	67889605	67895628	+
chr5	ENSG00000250874.1	ENST00000507387.1	85663232	85664684	+	85657594	85663641	−
chr5	ENSG00000250682.5	ENST00000510145.1	102609156	102671559	−	102671230	102677260	+
chr5	ENSG00000251026.1	ENST00000514769.1	104079911	104105403	+	104074363	104080381	−
chr8	ENSG00000253706.5	ENST00000518128.5	74816051	74866939	−	74866520	74872545	+
chr11	ENSG00000254444.1	ENST00000529961.1	6108135	6185576	−	6185351	6191366	+
chr11	ENSG00000254560.5	ENST00000530430.1	27063020	27220086	−	27219704	27225732	+
chr11	ENSG00000254746.5	ENST00000529127.5	45531216	45533534	−	45533534	45533886	+
chr11	ENSG00000254804.1	ENST00000528000.1	55684141	55686160	−	55685641	55691667	+
chr11	ENSG00000250519.6	ENST00000515097.2	94238150	94279206	+	94232525	94238528	−
chr12	ENSG00000258053.1	ENST00000549357.1	71047402	71118247	−	71118073	71124103	+
chr12	ENSG00000258815.1	ENST00000555596.1	85318060	85342912	+	85312420	85318459	−
chr14	ENSG00000257869.1	ENST00000548280.1	28318141	28418612	+	28312503	28318523	−
chr14	ENSG00000259129.5	ENST00000555985.5	47764954	47795014	−	47794966	47800647	+
chr14	ENSG00000266869.1	ENST00000555581.1	71848606	71908430	+	71842965	71848996	−
chr15	ENSG00000259692.5	ENST00000560054.5	81660482	81798124	−	81797931	81803963	+
chr15	ENSG00000259445.1	ENST00000559299.1	81953303	81995666	−	81995167	82001196	+
chr16	ENSG00000261310.1	ENST00000567862.1	60486819	60523250	−	60522746	60528760	+
chr16	ENSG00000261235.1	ENST00000567021.1	82044371	82139631	−	82139256	82145258	+
chr17	ENSG00000261848.5	ENST00000572821.5	3134969	3176935	−	3176531	3182557	+
chr18	ENSG00000266268.5	ENST00000584204.1	4807935	5004537	−	5004137	5010155	+
chr18	ENSG00000267413.1	ENST00000585822.1	39841174	39924840	−	39924370	39930434	+
chr18	ENSG00000267284.1	ENST00000587346.1	55721396	55784944	+	55715485	55721529	−
chr20	ENSG00000259723.1	ENST00000558738.1	53453058	53504314	−	53503852	53509874	+
chr21	ENSG00000237945.7	ENST00000427447.5	33931160	33977691	+	33925607	33931606	−
chrX	ENSG00000225882.1	ENST00000453902.1	17970197	18104644	−	18104293	18104924	+

A list of transcribed lncRNAs including antisense L1PA2 elements at the 5' ends is shown. Gene Id and Transcript Id are based on GENCODE v24 and represent each lncRNAs. LncRNA Start / End and L1PA2 Start / End show the coordinates (in hg38) of the lncRNAs and antisense L1PA2 elements.

Table S3: Samples used in the ChIP-seq analysis

**ID**	**Name**	**Cell group**
SRX038574	H3K4me3 (@HMEC)	Breast
SRX038594	H3K4me3 (@HSMM)	Muscle
SRX038614	H3K4me3 (@HUVEC)	Cardiovascular
SRX038656	H3K4me3 (@NHEK)	Epidermis
SRX038676	H3K4me3 (@NHLF)	Lung
SRX130264	H3K4me3 (@293)	Kidney
SRX186702	H3K4me3 (@Osteobl)	Bone
SRX190056	H3K4me3 (@HVMF)	Placenta
SRX190069	H3K4me3 (@HEEpiC)	Digestive_tract
SRX196106	H3K4me3 (@SGBS)	Adipocyte
SRX340783	H3K4me3 (@Pancreaticislets)	Pancreas
SRX481465	H3K4me3 (@Prostate)	Prostate
SRX663448	H3K4me3 (@Testis)	Gonad
SRX974429	H3K4me3 (@Liver)	Liver
SRX1096824	H3K4me3 (@OccipitalLobe)	Neural

Samples selected from the H3K4me3 histone modification data file (His.ALL.05.H3K4me3.AllCell.bed) downloaded from ChIP-Atlas

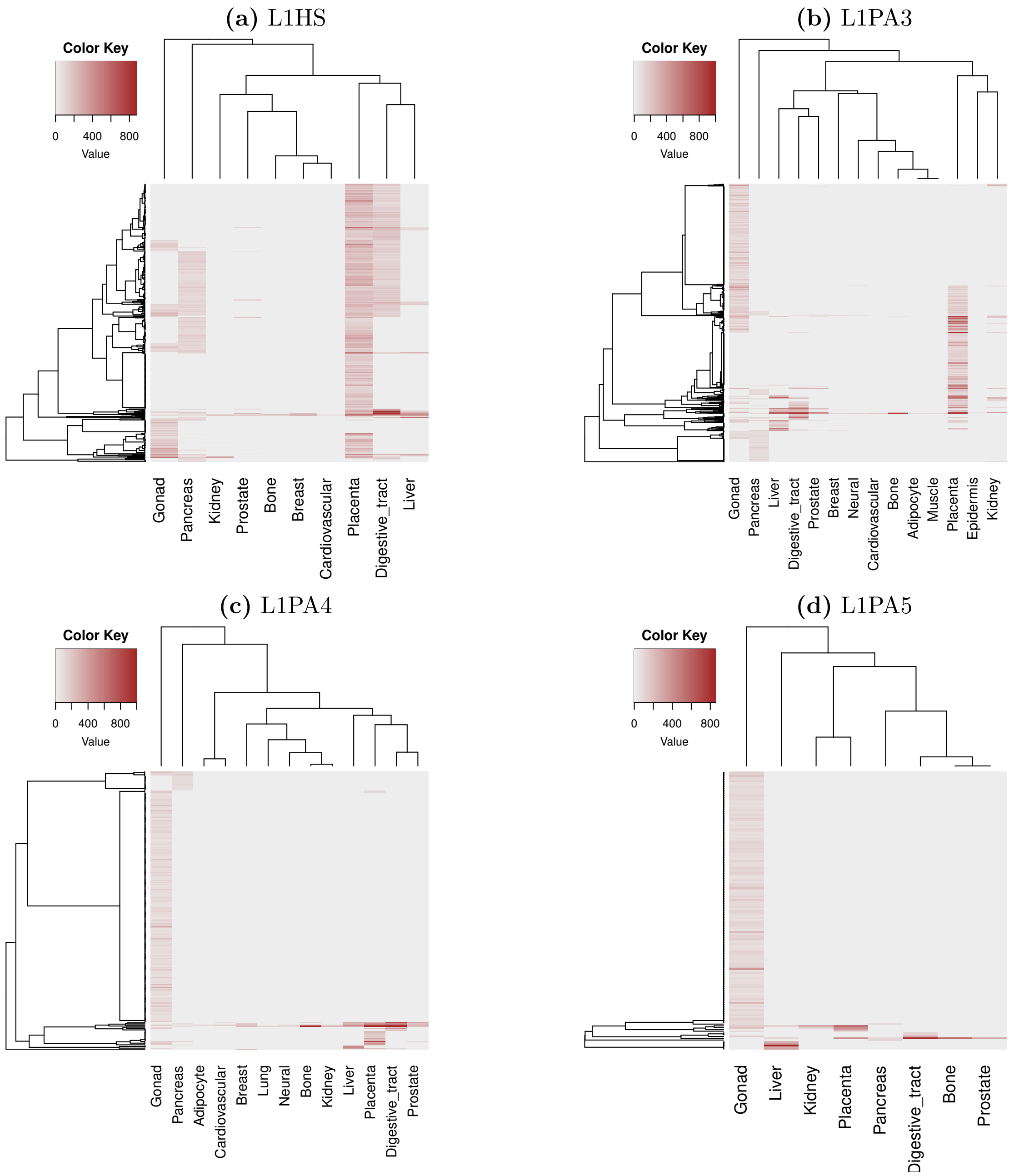

Figure S3: The H3K4me3 histone modification level of the each L1 subfamily transposable elements (TEs) closely related to L1PA2. Each row represents a specific L1 element, and each column represents a different sample. Only L1 elements overlapping peaks in one or more samples are shown. Samples in which all L1 elements did not overlap with peaks were excluded from the figure. The intensity of the color of each cell indicates the maximum value of the peak score ($-10\log(Q$-value)) within the 5' region (positions 0–1000) of each element. (If there are no peaks in the area, the score is 0.)
